# A Novel Blood-Brain Barrier-Permeable Chemotherapeutic Agent for the Treatment of Glioblastoma

**DOI:** 10.7759/cureus.17595

**Published:** 2021-08-31

**Authors:** Tomoko Ozawa, Mirna Rodriguez, Guisheng Zhao, Tsun Wen Yao, Wolf-Nicolas Fischer, Bernd Jandeleit, Kerry Koller, Theodore Nicolaides

**Affiliations:** 1 Neurological Surgery, University of California San Francisco, San Francisco, USA; 2 Science, Quadriga BioSciences, Los Altos, USA; 3 Pediatric Hematology-Oncology, New York University (NYU), New York, USA; 4 Science, Bristol Myers Squibb, San Diego, USA

**Keywords:** glioblastoma, qbs10072s, temozolomide, lat1, blood brain barrier

## Abstract

Introduction

The standard treatment for glioblastoma (GBM) patients is surgical tumor resection, followed by radiation and chemotherapy with temozolomide (TMZ). Unfortunately, 60% of newly diagnosed GBM patients express high levels of the DNA repair enzyme O6-methylguanine-DNA methyltransferase (MGMT) and are TMZ-resistant, and all patients eventually become refractory to treatment. The blood-brain barrier (BBB) is an obstacle to the delivery of chemotherapeutic agents to GBM, and BBB-permeable agents that are efficacious in TMZ-resistant and refractory patients are needed. The large amino acid transporter 1 (LAT1) is expressed on the BBB and in GBM and is detected at much lower levels in normal brain tissue. A LAT1-selective therapeutic would potentially target brain tumors while avoiding uptake by healthy tissue.

Methods

We report a novel chemical entity (QBS10072S) that combines a potent cytotoxic chemotherapeutic domain (tertiary N-bis(2-chloroethyl)amine) with the structural features of a selective LAT1 substrate and tested it against GBM models *in vitro* and *in vivo*. For *in vitro* studies, DNA damage was assessed with a gamma H2A.X antibody and cell viability was assessed by WST-1 assay and/or CellTiter-Glo assay. For *in vivo* studies, QBS10072S (with or without radiation) was tested in orthotopic glioblastoma xenograft models, using overall survival and tumor size (as measured by bioluminescence), as endpoints.

Results

QBS10072S is 50-fold more selective for LAT1 vs. LAT2 in transport assays and demonstrates significant growth suppression *in vitro* of LAT1-expressing GBM cell lines. Unlike TMZ, QBS10072S is cytotoxic to cells with both high and low levels of MGMT expression. In orthotopic GBM xenografts, QBS10072S treatment significantly delayed tumorigenesis and prolonged animal survival compared to the vehicle without adverse effects.

Conclusion

QBS10072S is a novel BBB-permeable chemotherapeutic agent with the potential to treat TMZ-resistant and recurrent GBM as monotherapy or in combination with radiation treatment.

## Introduction

Glioblastoma (GBM) is the most common primary malignant brain tumor in adults. Survival rates are dismal, and the only therapies shown to extend life, albeit briefly, are surgical resection, radiation therapy, and cytotoxic therapy with temozolomide (TMZ) in a subgroup of patients [1]. Numerous barriers exist to improved outcomes for patients with GBM, including the infiltrative nature of the disease, “cold” immune microenvironment [2], and the restrictive nature of the blood-brain barrier (BBB) to therapeutics.

The BBB is formed by tight junctions between the endothelial cells of the brain capillaries and restricts the passage of potentially damaging molecules into the brain. Attempts to bypass the BBB for improved drug delivery to GBM include convection-enhanced delivery, intranasal delivery, and BBB disruption. A novel approach would be to take advantage of active transporters throughout the BBB to transport toxic payloads directly into the tumor bulk and into tumor cells. One such transporter is the large neutral amino acid transporter 1 (LAT1; SLC7A5). LAT1 is expressed on both the luminal and abluminal membranes of the microvessels that form the BBB, facilitates the uptake of neutral amino acids, and has the greatest transport capacity among the BBB amino acid influx transporters [3-5]. LAT1 is responsible for the transport of central nervous system therapeutics, such as levodopa (L-DOPA) and gabapentin, across the BBB [6-7]. LAT1 has an oncofetal expression pattern and is overexpressed in most tumors, regardless of the tissue of origin [8-9]. Quantitative polymerase chain reaction (PCR) studies have shown a 40- to 400-fold overexpression of LAT1 in GBM compared to normal brain tissues [10]. LAT1 expression both in glioma cells and at the BBB also increases with tumor grade and stage in glioma patients [11].

We have generated a novel LAT1 substrate containing a cytotoxic chemotherapeutic moiety, which can transverse the BBB and enter into LAT1-expressing tumors to cause irreparable damage to the GBM cells. Our choice of cytotoxic agents is similar to melphalan, which has failed in GBM clinical trials possibly due to the ability of melphalan to be actively transported across the BBB less effectively than our compound. Additionally, our compound is predicted to not be susceptible to repair mechanisms performed by O6-methylguanine-DNA methyltransferase (MGMT) due to its ability to cause both DNA crosslinking and DNA alkylation. In this manuscript, we present *in vitro* and *in vivo* data supporting the use of this molecule as a single agent and in combination with radiation therapy against GBM.

## Materials and methods

QBS10072S synthesis

QBS10072S ((+)-(3S)-amino-4-[5-[bis(2-chloroethyl)amino]-2-methyl-phenyl]butanoic acid, CAS RN: 1802735-28-9) was prepared in six synthetic steps as described [12]. [14C]-QBS10072S ([chloroethyl- 2-14C]-QBS10072S×2HCl) was prepared using 2-[14C]-chloroacetic acid following the above procedure and had a molar activity of 37.4 mCi/mmol and a radiochemical purity of 95% (Moravek, Brea, CA).

Cell lines

LLC-PK1 cell lines expressing either hLAT1 or hLAT2 under the control of a tetracycline-inducible promoter were obtained as a gift from XenoPort, Inc. (Santa Clara, CA). Xenoport used the Invitrogen Gateway pT-REx System to develop the expression plasmid. 3T3 fibroblasts, normal human astrocytes (NHA), and human GBM cell lines (including AM38, DBTRG, U251, LN229, 8MGBA, 42MGBA, U138, NMCG1, U87, and U87:MGMT) were obtained from Dr. Russ Pieper at UCSF. SF7183 and SF188 cells were generated from patient samples under UCSF IRB NUMBER: 10-01318. Cells were propagated in high glucose DMEM with 10% FBS at 37°C in an atmosphere of 5% CO2. All cell lines were authenticated using DNA fingerprinting (Promega platform) and compared to in-house and published databases.

Transport uptake assays

Substrate transport inhibition studies were conducted with LLC-PK1 cells inducibly expressing either hLAT1 or hLAT2. Cells were plated in growth media with 1 μg/mL doxycycline at 30,000 cells/well in 96-well plates. Sixteen hours after plating, cells were treated with butyrate at a final concentration of 5 mM. The following day, cells were washed with PBS and incubated in the presence or absence of various concentrations of QBS10072S with 30,000 cpm/well of either [3H]-gabapentin (LAT1) or [3H]-leucine (LAT2) in PBS for 15 minutes at room temperature. The non-specific cell-bound activity was determined by the addition of 5 mM unlabeled gabapentin (LAT1) or leucine (LAT2). Unincorporated radioactivity was removed, and cells were washed three times with ice-cold PBS. Cells were lysed by adding scintillation fluid to each well, and cell-associated radioactivity was determined via scintillation counting using a multichannel 96-well plate reader (MicroBeta Trilux, Perkin Elmer, Waltham, MA). Data were expressed as the percent of specific radioactivity uptake in wells with no compound added. LC50s were determined by non-linear curve-fitting using GraphPad Prism (San Diego, CA).

*In vitro* cell viability assays

*In vitro* activity of QBS10072S was assessed using a modified clonogenic cell viability assay in 96-well clear-bottom plates. For the LLC-PK1 inducible LAT1-expressing cell line, 1000 cells/well were plated and cells were treated with either butyrate alone (non-induced) or with both butyrate and doxycycline (induced) as described above. For GBM cell lines, 2000 cells/well were plated. On day 2 (GBM) or day 3 (LLC-PK1) after original plating, cells were washed twice with PBS and incubated with 50 μL of various concentrations of QBS10072S in PBS in at least quadruplicate. After 30 minutes at 37°C, 200 μL of growth medium was added to each well. Clonal populations were allowed to grow until the control wells (mock treatment) were near confluence (7 to 10 days). At this point, 170 μL media were removed and 60 μL CellTiter-Glo® (Promega, Madison, WI) were added to each well. Plates were placed on an orbital shaker for three minutes to lyse the cells and then incubated for an additional 10 minutes at RT to stabilize the luminescent signal. Luminescence was recorded using an EnSpire® Multimode Plate Reader (Perkin Elmer). Background (luminescence from wells incubated with 100 μM melphalan or lomustine) was subtracted and data were normalized to the control. EC50s were determined by non-linear regression using GraphPad Prism.

WST1 cell viability assays of QBS10072S and TMZ in U87 and U87-MGMT cells

*In vitro* activities of QBS10072S and temozolomide (TMZ) in U87 and U87-MGMT cells were assessed using the WST1 cell viability assay in 96-well plates in which 500/well of cells were plated in sextuplets for each condition. To avoid cell loss, the following washing and medium removing procedures were all done using a P200 pipet without vacuum aspiration. For QBS10072S treatment, cells were washed with PBS the next day and incubated with 50 μL of various concentrations of QBS10072S in PBS for 1 hour at 37°C. After incubation, 150 μL of growth medium was added to each well. The medium was subsequently changed every two days until day 7. For TMZ treatment, cells were treated with 100 μL of various concentrations of TMZ in a growth medium for 2 days. After incubation, cells were put back in a normal growth medium and continued culturing for five days. In the end, 10 μL of WST1 reagent was added to each well and incubated at 37°C for 4h before subjecting to plate reading at 450 nm and 690nm using an EnSpire multimode plate reader (PerkinElmer). Background (OD690nm) was subtracted and data were normalized to the control. LC50s were determined by non-linear regression using GraphPad Prism (GraphPad Software Inc., San Diego, California)

Phosphorylation of γ-H2AX assay

U251 or normal human astrocyte cells were plated in six-well plates and incubated with various concentrations of QBS10072S for 16 hours. Proteins were extracted from cells using cell lysis buffer (Cell Signaling Technologies, Danvers, MA) supplemented with proteinase and a phosSTOP phosphatase inhibitor cocktail (Sigma-Aldrich, St. Louis, MO). Proteins were separated by SDS-PAGE and transferred onto polyvinylidene difluoride membranes, which were then probed with primary antibodies followed by horseradish peroxidase-conjugated secondary antibody, and visualized by ECL (Thermo-Fischer, S. San Francisco, CA). Antibodies specific for γH2AX (#2577), LAT1 (#5347), and beta-actin (#4970) were obtained from Cell Signaling Technologies.

Intracranial tumor establishment in athymic mice

Five-week-old female athymic mice (nu/nu, homozygous: Simonsen Laboratories, Gilroy, CA), housed under aseptic conditions, received intracranial tumor cell injection as previously described [13] and as approved by the University of California San Francisco Institutional Animal Care and Use Committee, Protocol #: AN111064-03. Briefly, mice were anesthetized by intraperitoneal injection of ketamine (100 mg/kg) and xylazine (10 mg/kg) and injected with 3 μL of tumor cell suspension (300,000 cells total) into the right caudate-putamen. Coordinates: 3 mm to the right from bregma, on top of the coronal suture, and 3.5 mm depth. Injection rate: ~30 second/uL. For treatment studies, eight mice were used for each group for LN229 studies and nine mice per group for U251 studies. Treatments began when tumors entered the log-phase of growth as measured by luminescence, for the LN229 experiment on Day 21 and for U251 experiments on Day 14.

QBS10072S dosing into tumor-bearing mice

QBS10072S was dosed intravenously (IV) into the tail vein with a dosing volume of 7.5 mL/kg using a formulation prepared in 94:6 (v/v) 50 mM sodium citrate buffer pH 8.0 and a solubilizing mixture (57.5% 1,2- propylene glycol; 30% Kolliphor HS-15; 12.5% ethanol). Dosing levels and frequency are described in the figure legends.

[14C]-QBS10072S distribution in tumor-bearing mice

Athymic mice with intracranial implanted LN229 cells received a single IV dose of 5 mg/kg and 200 μCi/kg [14C]-QBS10072S into the tail vein using the formulation described above. Animals (one per time point) were sacrificed one, eight, and 24 hours post-dose, and frozen in a hexane/solid carbon dioxide bath for at least 10 minutes. The carcasses were processed as described [14] for quantitative whole-body autoradiography. Briefly, sagittal sections (~ 40 μm thick) were cut using a microtome, mounted, and exposed to a [14C]-sensitive phosphor imaging plate for four days. The images were scanned and quantitated by image densitometry using MCID image analysis software. The concentrations of radioactivity were expressed as the μg equivalents of test article per gram sample (μg equiv/g). The upper and lower limits of quantification were 550 and 0.008 μg equiv/g, respectively (QPS, Newark, DE).

Bioluminescence monitoring of intracranial tumor growth

For bioluminescence imaging (BLI), mice were anesthetized as above, then administered 150 mg/kg of luciferin (D-luciferin potassium salt, Gold Biotechnology, St. Louis, MO) via intraperitoneal injection. Ten minutes after luciferin injection, mice were examined for tumor bioluminescence with an IVIS Lumina imaging station and Living Image software (Caliper Life Sciences, Alameda, CA), and regions of interest were recorded as photons per second per steradian per square cm [13].

Mouse irradiation

Mice were anesthetized via inhalation of 2.5% isoflurane with 1 L of oxygen per minute for five minutes prior to being positioned on an irradiation platform located 16.3 cm from a cesium-137 source (JL Shepherd & Associates, San Fernando, CA). Eyes, respiratory tracts, and bodies were protected with lead shielding. Mice received whole-brain irradiation at a dose rate of 247 cGy/min until 1.5 Gy radiation had been administered [15]. After irradiation, animals were monitored until recovery. Mice were irradiated once daily for five consecutive days. All mice received the first radiation dose when tumors were in a log-phase growth (Day 14) as determined by BLI.

Temozolomide dosing in tumor-bearing mice

Tumor-bearing mice were treated with temozolomide (TMZ) 4 mg/kg via oral gavage, starting on day 14 post U251 cell injection once daily for five days - concurrent therapy with RT. TMZ was obtained from the UCSF hospital pharmacy and formulated with the Ora-Plus suspending vehicle (Perrigo, Dublin, Ireland) with a working concentration of 1 mg/mL.

Statistical analysis

PRISM 5, Version 5.03 (GraphPad) was used to conduct all statistical analyses. For survival analysis, significance was determined by the log-rank (Mantel-Cox) test.

## Results

Characterization of QBS10072S LAT1-mediated activity *in vitro*

**Figure 1:**
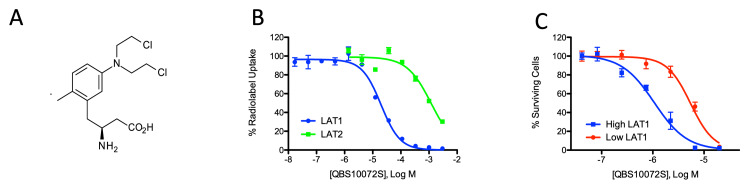
LAT1 selectivity of QBS10072S A: Chemical structure of QBS10072S; B: Inhibition of radiolabeled substrate uptake by QBS10072S into LAT1 or LAT2 expressing LLC-PK1 cell lines. Cells were incubated with various concentrations of QBS10072S with either 3H-gabapentin (LAT1) or 3H-leucine (LAT2), and IC50 values were determined as described in the methods; C: Effect of LAT1 expression on QBS10072S-induced cell viability loss. LLC-PK1-LAT1 cells were induced (high LAT1) or not induced (low LAT1) to express LAT1. Cells were incubated with a titration of QBS10072S and cell viability was measured by CellTiter-Glo. EC50 values for each condition were determined.

QBS10072S is a dual-function compound with a LAT1-targeting element and a tertiary bis(2-chloroethyl)amine cytotoxic moiety (Figure 1A). LAT2 is a structurally related amino acid transporter to LAT1. We tested the selectivity of QBS10072S using an isogenic LLC-PK1 cell pair that expresses either LAT1 or LAT2 under the control of a tetracycline-inducible promoter. QBS10072S inhibits substrate transport in high LAT1-expressing cells 50-fold more potently compared to LAT2-expressing cells (IC50s = 21 μM vs. 1100 μM, respectively; Figure 1B). QBS10072S was 5.5 times more potent at suppressing viability in high LAT1 expressing cells (induced; EC50 = 1.0 μM) vs. non-induced cells (EC50 = 5.5 μM), which had low, endogenous levels of LAT1 expression (Figure 1C). These results suggest that QBS10072S is preferentially transported into cells expressing LAT1 where it exerts its cytotoxic effect.

**Figure 2:**
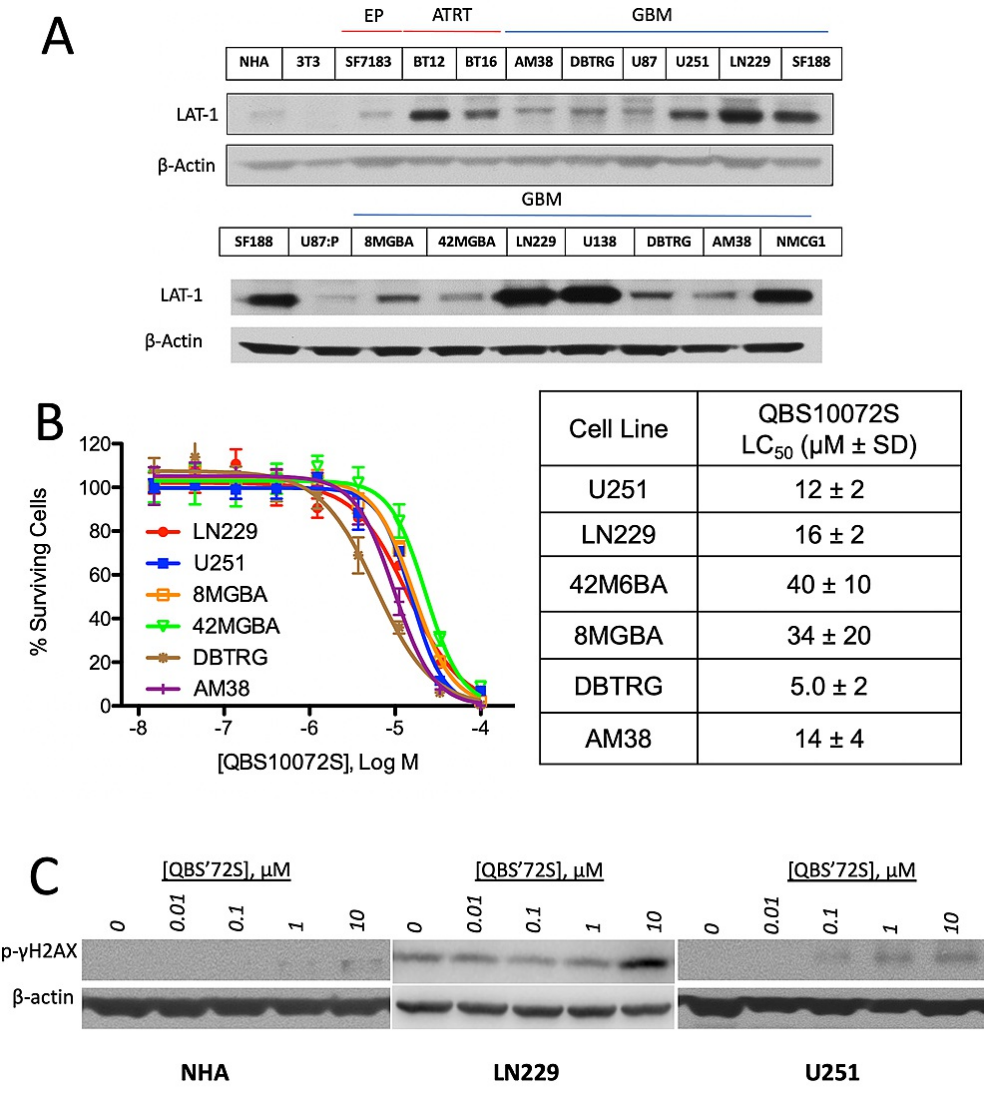
LAT1 expression and in vitro cytotoxicity of QBS10072S in GBM cell lines A: LAT1 expression in various GBM cell lines as determined by western analysis. B: *In vitro* cytotoxicity of QBS10072S in various GBM cell lines. C: QBS10072S induction of DNA damage repair (DDR) pathway in GBM (LN229, U251) vs. normal human astrocyte (NHA) cells. Dose-dependent phosphorylation of H2AX in GBM cells but not normal astrocytes. Cells were treated with a titration of QBS10072S for 16 hours. Phospho-H2AX levels were assessed by immunoblot analysis. GBM: glioblastoma

**Figure 3:**
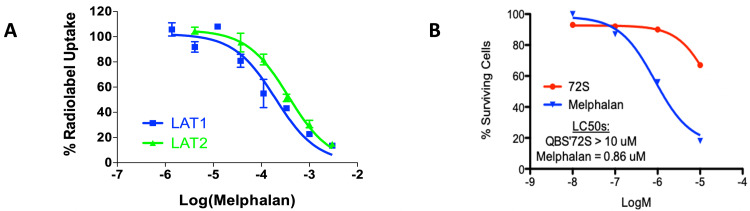
Melphalan activity on LAT transporters and normal human astrocytes A: Inhibition of radiolabeled substrate uptake by melphalan into LAT1 or LAT2 expressing LLC-PK1 cell lines. Cells were incubated with various concentrations of melphalan with either 3H-gabapentin (LAT1) or 3H-leucine (LAT2) and IC50 values were determined as described in the methods; B: Cytotoxicity of QBS10072S vs. melphalan in normal human astrocytes. Cells were incubated with a titration of QBS10072S or melphalan and cell viability was measured by CellTiter-Glo. LC50 values for each compound were determined. Melphalan is less active on LAT1 than QBS10072S and is less selectivity with higher activity on LAT2 than QBS10072S. Melphalan shows higher cytotoxicity to NHA cells than QBS10072S.

LAT1 is expressed highly in numerous tumors types including GBM [11,16-19] (Figure 2A). In contrast, normal human tissues, such as normal human astrocytes (NHAs), express low levels of LAT1 (Figure 2A).* In vitro *studies on multiple GBM cell lines showed that QBS10072S induced dose-dependent viability loss with EC50s ranging from 12 to 40 μM (Figure 2B). High LAT1 expressing GBM cell lines, U251 and LN229, were among the most sensitive to QBS10072S and were therefore selected for subsequent *in vivo *studies. In contrast, the chemotherapeutic agent melphalan is transported less efficiently by LAT1 and less selectively across cell membranes by using multiple transporters than QBS10072S (Melphalan IC50s = 170 and 390 μM for LAT1 and LAT2, respectively). Melphalan also significantly decreases viable normal human astrocyte cell numbers at lower doses compared to the LAT1-selective QBS10072S (Figure 3).

Cytotoxic compounds such as tertiary bis(2-chloroethyl)amine alkylating agents induce cell death through DNA damage, and the activity of these cytotoxic agents can be assessed by measuring the activation of DNA damage repair (DDR) pathways. A well-established approach of detecting early DNA double-strand breaks is through measuring phosphorylation of the Ser-139 residue of the histone variant H2AX, which leads to the formation of γ -H2AX [20]. QBS10072S treatment demonstrated dose-dependent phosphorylation of H2AX in U251 and LN229 GBM cells (Figure 2C), indicating compound-induced DNA damage and confirming the mechanism of QBS10072S. In contrast, a significantly lower DDR pathway activation was seen in NHAs where basal LAT1 was undetectable. Collectively, these data support our hypothesis that QBS10072S is transported into LAT1-expressing GBM cells where it causes dose-dependent DNA damage that leads to cell death.

**Figure 4:**
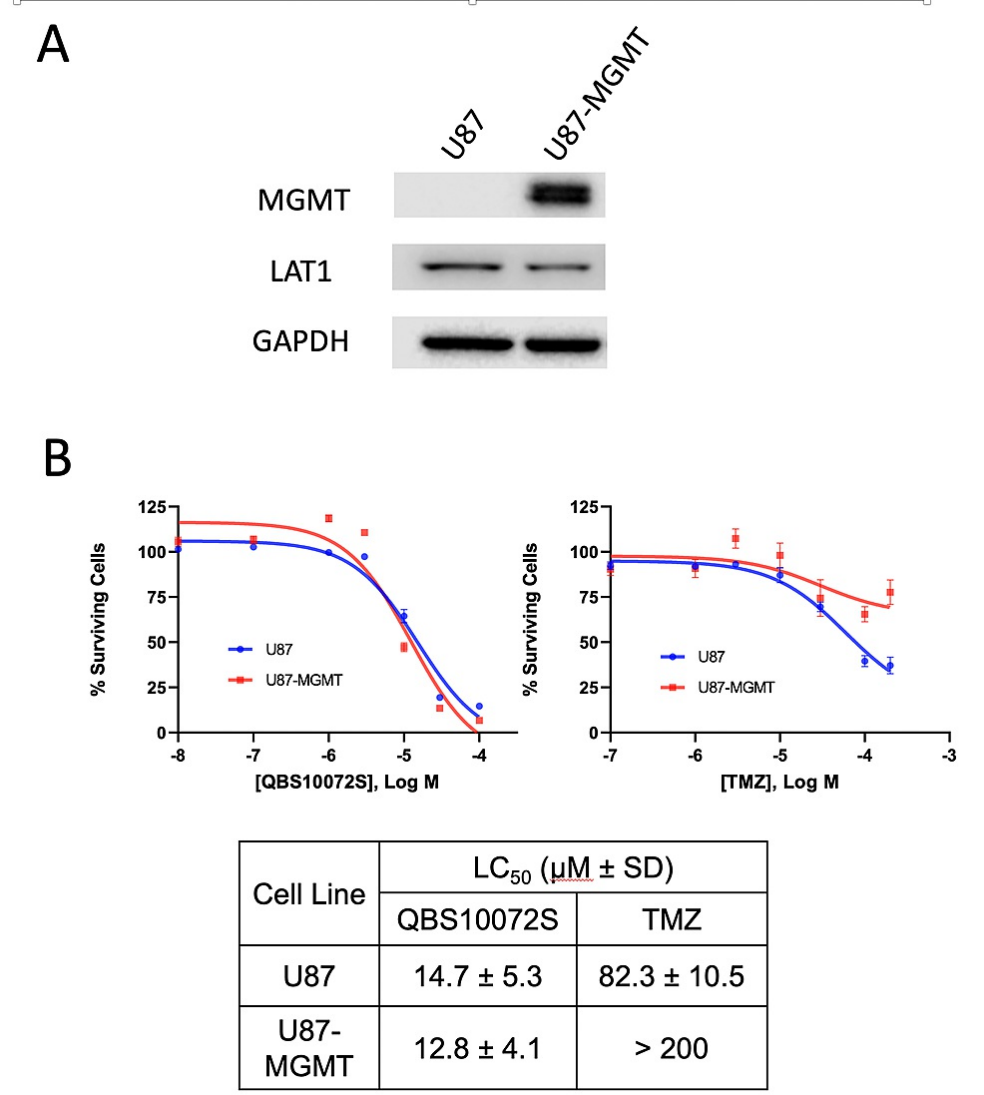
In vitro cytotoxicity of QBS10072S independent of MGMT expression status in glioma cell line U87 A: Western analysis of LAT1 and MGMT expression in both cell lines. B: Effect of QBS10072S (left) vs. TMZ (right) on cell viability in U87 cells with and without the expression of the DNA repair enzyme MGMT. Cells were treated with increasing concentrations of QBS10072S or TMZ, and cell viability was determined as described in methods. LC50 values from dose-response curves are listed.

Many GBM patients are resistant or become resistant to TMZ. One aspect of this resistance is mediated by the expression of O6-methylguanine-DNA methyltransferase (MGMT), an enzyme that can repair the DNA damage caused by TMZ [1]. Since QBS10072S and TMZ have distinct mechanisms of cell killing, we hypothesized that the cytotoxicity of QBS10072S would be independent of the expression of MGMT. We used an isogenic glioblastoma system (U87) that was modified to overexpress MGMT [21] (Figure 4A) to investigate the effects of TMZ versus QBS10072S. As shown in Figure 4B, QBS10072S had similar effects on cell viability in the U87 parental and MGMT overexpressing cells. In contrast, TMZ was significantly less effective in the MGMT overexpressing cells. These results suggest that QBS10072S may be potent at inhibiting tumor cell growth in patients whose tumors express MGMT and do not respond to TMZ.

Biodistribution of [14C]-QBS10072S

**Figure 5:**
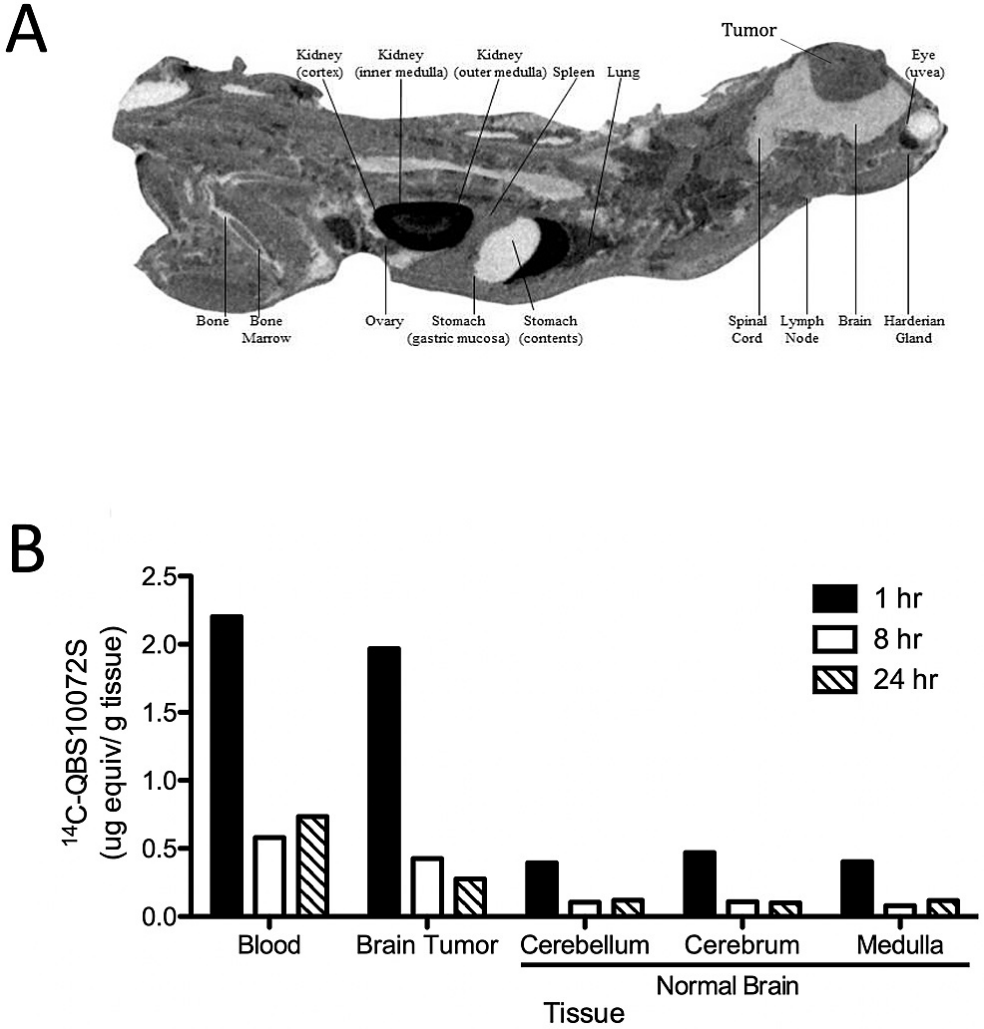
Distribution of [14C]-QBS10072S Biodistribution of [14C]-QBS10072S in an orthotopic mouse GBM model. Mice with intracranially implanted LN229 cells were treated with a single IV dose of 5 mg/kg and 200 µCi/kg [14C]-QBS10072S. A: Whole-body quantitative autoradiogram of radioactivity distribution one-hour post-dose. The mouse was sacrificed and examined as described in methods; B: Radioactivity measured in blood vs. tumor vs. normal brain expressed as µg equivalent/ g tissue at one, eight, and 24 hours post-dose. One animal was used for each time point.

The biodistribution of radiolabeled QBS10072S was determined by quantitative whole-body autoradiography (QWBA) after dosing 5 mg/kg and 200 μCi/kg of [14C]-QBS10072S into animals with orthotopic brain tumors derived from LN229 cell implantation. Radioactivity throughout the body was measured one, eight, and 24 hours post-dosing (Table 1). As expected, high levels of radioactivity were detected in excretory organs such as the kidney and gastrointestinal tract. Notably, QBS10072S showed favorable BBB penetrance and was retained in the brain tumor without significant accumulation in the surrounding normal brain tissue (Figure 5A). Levels of QBS10072S in the brain tumor were 89%, 73%, and 37% of blood levels at one, eight, and 24 hours post-dose, respectively, while the radioactivity in the surrounding normal brain was 13%-20% of blood levels at all time points (Figure 5B).

**Table 1:** [14C]-QBS10072S levels in tissues as determined by quantitative whole-body autoradiography (QWBA) LLOQ = 0.0003 µCi/g ÷ 0.0380645 µCi/µg = 0.008 µg equivalent/g tissue ULOQ = 20.9730 µCi/g ÷ 0.0380645 µCi/µg = 550.986 µg equivalent/g tissue

			Tissue Concentration (µg equiv/g tissue)				
Tissue Type		Tissue	1 h	8 h		24 h	
Vascular/Lymphatic		Blood (cardiac)	2.204	0.581		0.734	
		Bone Marrow	1.320	0.386		0.520	
		Lymph Node	1.670	0.481		0.354	
		Spleen	1.625	0.370		0.503	
Excretory/Metabolic		Bile (in gall bladder)	3.649	2.006		0.564	
		Kidney (cortex)	5.848	1.272		1.077	
		Kidney (inner medulla)	3.704	0.498		0.458	
		Kidney (outer medulla)	6.281	1.372		1.164	
		Liver	5.522	0.674		0.598	
		Urinary Bladder	3.914	0.821		0.485	
		Urinary Bladder (contents)	206.798	1.716		0.811	
Central Nervous System		Brain (cerebellum)	0.395	0.105		0.120	
		Brain (cerebrum)	0.469	0.109		0.101	
		Brain (medulla)	0.403	0.081		0.118	
		Tumor	1.961	0.427		0.275	
		Tumor High	2.836	0.575		0.479	
		Tumor Low	1.245	0.162		0.240	
		Choroid Plexus	0.815	0.159		0.186	
		Spinal Cord	0.480	0.100		0.120	
Endocrine		Adrenal Gland	2.700	0.638		0.533	
		Pituitary Gland	1.689	0.468		0.128	
		Thyroid	1.802	0.374		0.653	
Secretory		Harderian Gland	3.371	0.468		0.329	
		Mammary Gland Region	0.485	0.173		0.365	
		Pancreas	3.270	0.546		0.442	
		Salivary Gland	2.429	0.428		0.368	
Fatty		Adipose (brown)	2.528	0.440		0.577	
		Adipose (white)	0.409	0.348		0.381	
Dermal		Skin	1.496	0.479		0.425	
Reproductive		Ovary	2.564	0.563		0.585	
		Uterus	1.849	0.563		0.511	
		Vagina	1.337	0.640		0.629	
Skeletal/Muscular		Bone	0.350	0.165		0.065	
		Heart	2.165	0.489		0.480	
		Skeletal Muscle	1.123	0.602		0.627	
Respiratory Tract		Lung	2.943	0.684		0.620	
Alimentary Canal	Cecum		2.419		0.924		0.655
	Cecum (contents)		3.895		24.266		9.502
	Esophagus		1.211		0.442		0.565
	Large Intestine		1.437		0.594		0.511
	Large Intestine (contents)		4.090		147.823		19.396
	Oral Mucosa		0.900		0.399		0.390
	Small Intestine		4.374		0.760		0.355
	Small Intestine (contents)		65.367		1.429		3.802
	Stomach (gastric mucosa)		1.066		0.591		0.565
	Stomach (contents)		0.160		0.405		4.882
Ocular	Eye (lens)		0.078		0.023		0.013
	Eye (uvea)		0.692		0.277		0.166

Tumor suppressive activity of QBS10072S* in vivo*

**Figure 6:**
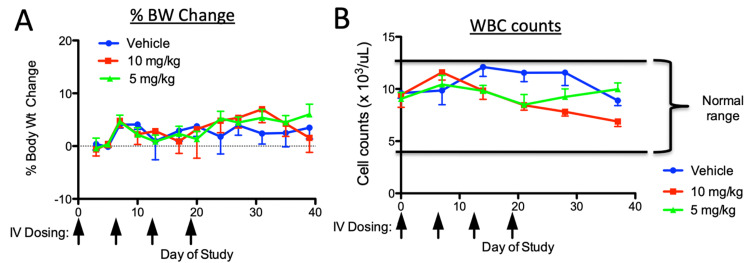
Tolerability of QBS10072S in nude mice Non-tumor-bearing nude mice were dosed IV with vehicle or QBS10072S (5 or 10 mg/kg) once a week for four weeks (arrows). Body weights were measured every three days and complete blood counts were determined once a week for seven weeks. A: Percent change in body weight over the course of the study, normalized to pre-dose weight. B: White blood cell (WBC) counts for each study arm. Horizontal bars represent the normal range of WBCs in these mice.

**Figure 7:**
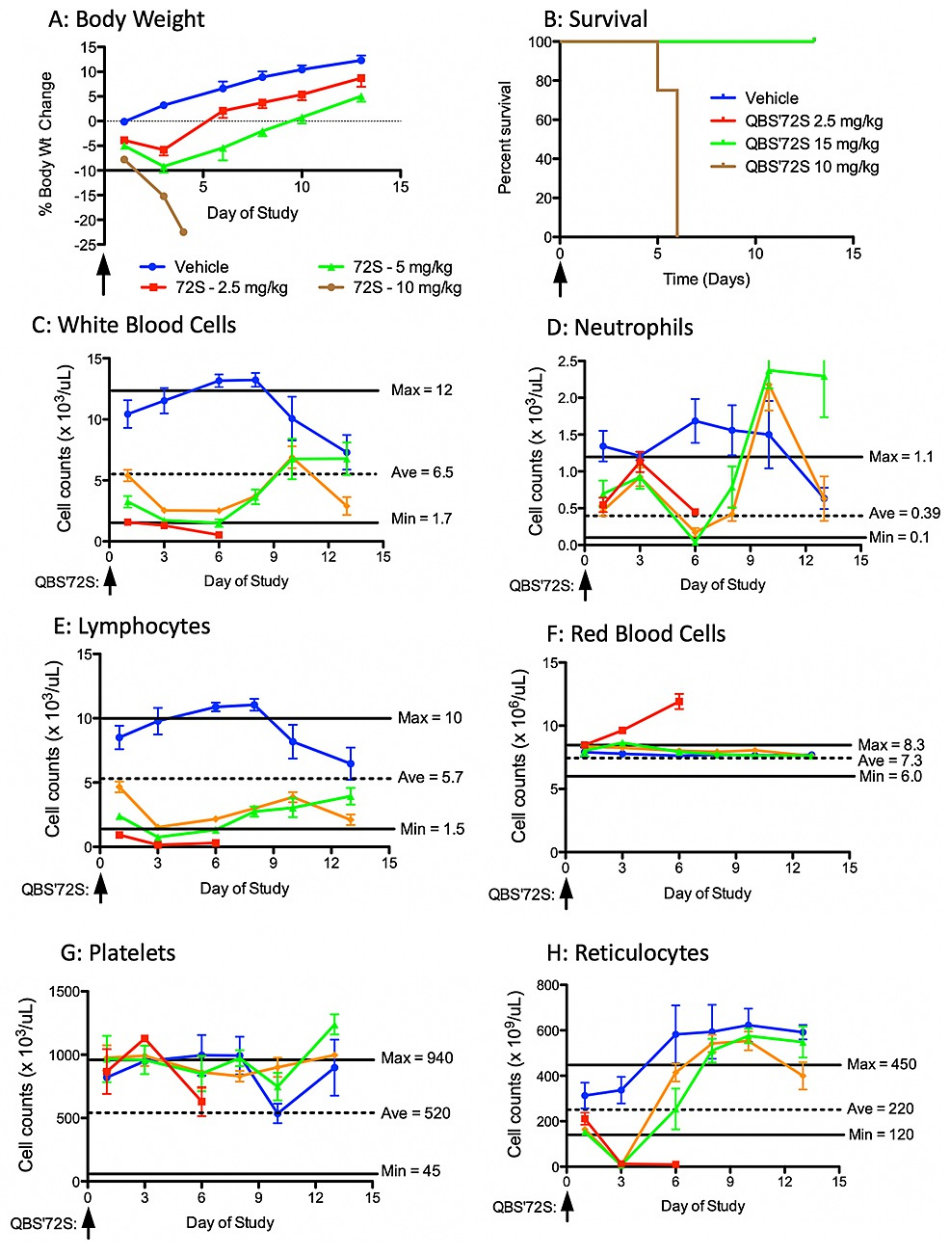
Maximum tolerated dose in rats Sprague-Dawley rats (4 per arm) were dosed IV on day zero with vehicle or 2.5, 5, or 10 mg/kg of QBS10072S. Animals were monitored for two weeks for body weight changes (A), survival (B), and complete blood counts, including white blood cells (C), neutrophils (D), lymphocytes (E), red blood cells (F), platelets (G), and reticulocytes (H). Cell count results are expressed as cell count per μl. The normal cell count range for each type of cell is between the “max” and “min” lines, and the average value is indicated with a dotted line. Based on allometric scaling, 2.5, 5, and 10 mg/kg in rats is equivalent to 5, 10, and 20 mg/kg. In these studies, the highest dose was greater than the maximum tolerated dose, which is consistent with the studies in mice.

The tumor-suppressive activity of QBS10072S was evaluated in two orthotopic murine models of GBM using luciferase-expressing U251 and LN229 cell lines. Tumor volume was monitored by BLI measurement. The tolerability of QBS10072S was first tested by dosing 5 or 10 mg/kg IV once a week for four weeks into non-tumor-bearing nude mice (Figure 6). The body weights and white blood cell counts were not affected by this treatment, so QBS10072S was tested at various doses up to 10 mg/kg in the GBM models. More extensive toxicity studies in rats showed similar results (Figure 7).

**Figure 8:**
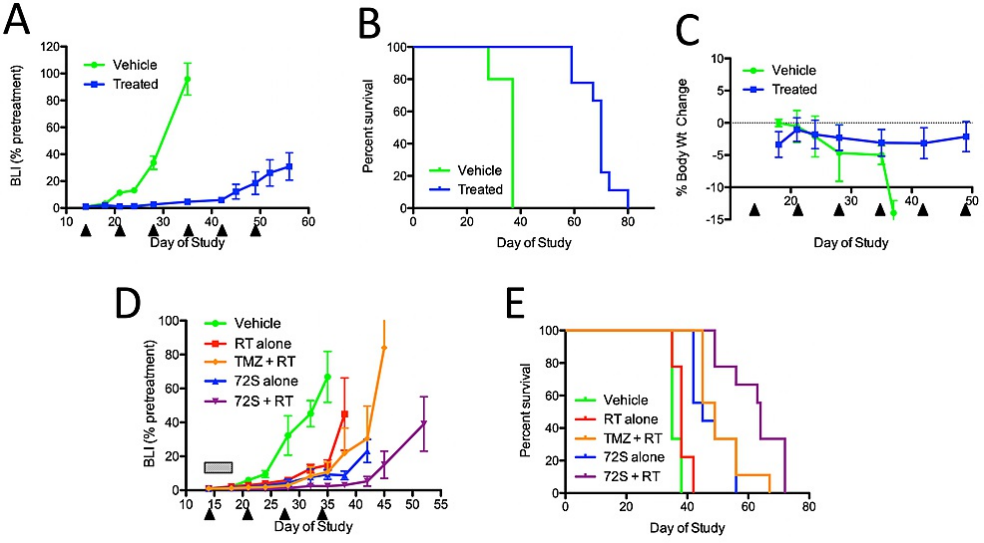
Activity of QBS10072S in the U251 intracerebral human GBM xenograft A: Tumor growth as measured by BLI after treatment with the vehicle or 10 mg/kg QBS10072S. The compound was dosed IV once a week for six weeks as indicated by the arrowheads. Data were expressed as average BLI normalized to pretreatment +/- SEM. B: Kaplan-Meier survival curves for both study arms. C: Percent body weight change across the course of the study. D: Tumor growth as measured by BLI after treatment with vehicle, radiation therapy (RT; 1.5 Gy daily for 5 days, hatched box), TMZ (4 mg/kg via oral gavage daily for 5 days) + RT (1.5 Gy daily for 5 days), QBS10072S alone (10 mg/kg IV once weekly for 4 weeks, arrowheads), or a combination of RT and QBS10072S. E: Kaplan-Meier survival curves for all study arms. BLI: bioluminescence imaging; TMZ: temozolomide; RT: radiotherapy

QBS10072S significantly inhibited tumor growth in the U251 orthotopic model (Figure 8A). The compound was dosed at 10 mg/kg IV once a week for six weeks. On day 35 of the study, the last day, the majority of the vehicle-treated animals were still alive, treatment with QBS10072S showed significant tumor growth inhibition (TGI) vs. vehicle treatment (TGI = 95%; p < 0.0001). Survival of the treated animals also increased, with median survival times of 37 vs. 70 days for a vehicle vs. QBS10072S-treated animals, respectively (p < 0.0001; Figure 8B). As expected, QBS10072S was well-tolerated with no significant body weight loss over the course of the study (Figure 8C). Since the standard of care for treating GBM patients includes radiotherapy (RT) and temozolomide, the combination of QBS10072S (10 mg/kg once a week for 4 weeks) and RT (1.5 Gy once daily for 5 days) was evaluated (Figure 8D). While treatment with RT or QBS10072S alone significantly inhibited tumor growth compared to vehicle control (%TGI at day 35 of 78% and 86%, respectively; p < 0.005), the effect of RT and QBS10072S combination treatment was more robust than single therapies (%TGI at day 35 = 96%; p < 0.001 vs. vehicle; p < 0.005 or 0.05 vs. RT or QBS10072S alone, respectively). The median survival time also increased significantly with combination therapy (vehicle: 35 days; RT alone: 38 days; QBS10072S alone: 45 days; QBS10072S + RT: 64 days; Figure 8E). The combination of QBS10072S and RT was also superior to temozolomide plus RT (median survival time of 48 days). Therefore, QBS10072S is potentially a complementary therapy for GBM patients who have received RT.

**Figure 9:**
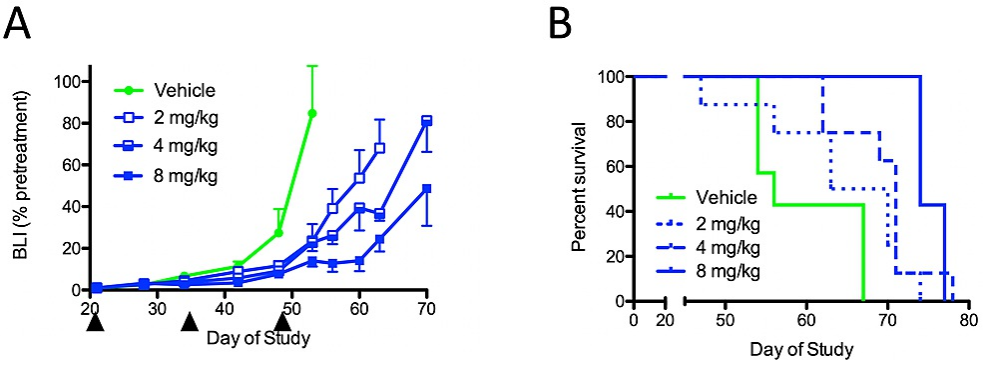
Activity of QBS10072S in the LN229 intracerebral human GBM xenograft A: Tumor growth as measured by BLI after treatment with vehicle or 2, 4, or 8 mg/kg QBS10072S. The compound was dosed IV a total of three times once every other week as indicated by the arrowheads. Data were expressed as average BLI normalized to pretreatment +/- SEM. B: Kaplan-Meier survival curves for all study arms. BLI: bioluminescence imaging

A less frequent dosing regimen (once every two weeks) was evaluated in a second orthotopic GBM model using LN229 cells. Multiple-dose levels of QBS10072S (2, 4, or 8 mg/kg) were compared. Dose-dependent suppression of tumor growth was observed. At all dose levels tested, significant %TGI was seen at day 53 (72% & 73% at 2 & 4 mg/kg, respectively, p < 0.05 vs. vehicle; 84% at 8 mg/kg, p < 0.01 vs. vehicle; Figure 9A). Median survival time extended significantly in subjects treated with 4 mg/kg or higher (vehicle: 56 days; 2 mg/kg: 66.5 days; 4 mg/kg: 71 days, p < 0.005; 8 mg/kg: 74 days, p < 0.01; Figure 9B). The data from two independent intracranial human GBM xenografts show that QBS10072S is efficacious in suppressing GBM growth and can be administered with a biweekly regimen.

## Discussion

The current standard of care for patients with GBM is surgical resection as a first-line treatment; however, since the tumor is rarely completely removed or maybe in portions of the brain that are not amenable to surgery, the residual tumor is treated with radiation and the chemotherapeutic agent TMZ. TMZ is an alkylating agent that nonspecifically crosses the BBB and other membranes via passive mechanisms, which can lead to dose-limiting toxicities to off-target tissues. The primary cytotoxic mechanism of TMZ is via alkylation of the O6 position of the DNA base residue guanine, which leads to the formation of lethal base-pair mismatch and subsequent tumor cell apoptosis. Unfortunately, 60% of newly diagnosed GBM patients do not respond to TMZ [1] and all patients eventually become refractory to treatment. Chemo-resistance in patients who do not respond to TMZ is mainly caused by the expression and activity of the DNA repair enzyme MGMT, which removes the chemotherapy-induced methyl group, thus preventing the DNA damage that leads to tumor cell death. MGMT-mediated repair is the most important factor responsible for resistance to TMZ chemotherapy in the treatment of GBM. MGMT expression is regulated by the methylation of its promoter; hypermethylation prevents the transcription of the MGMT gene leading to low expression of the MGMT repair enzyme. Consistent with the importance of this repair activity, MGMT promoter methylation status in GBM patients is strongly correlated with overall outcome [1].

Melphalan, bendamustine, and chlorambucil belong to a class of bifunctional, tertiary bis(2-chloroethyl)amine alkylating agents and are associated with extensive and durable DNA damage. They preferentially alkylate DNA at the N7 position of guanine and the N3 position of adenine, resulting in the formation of mono-adducts and intra- and inter-strand crosslinks (ICLs). ICLs distort the DNA double helix and cause the termination of DNA replication and the generation of double-strand breaks near the cross-linked site, leading to critical cytotoxic lesions if not repaired [22]. The cytotoxic potential of tertiary bis(2-chloroethyl)amine drugs is much higher than mono-alkylators such as TMZ due to the ability of tertiary bis(2-chloroethyl)amine drugs to form lethal ICLs [23]. The increased potency of tertiary bis(2-chloroethyl)amine drugs may also be due to secondary mechanisms such as inhibition of mitotic checkpoints, inefficient DNA repair, and initiation of p53-dependent DNA damage stress response, all of which lead to mitotic catastrophe and apoptosis. At least two DNA repair pathways are known to be involved in the removal of ICLs: non-homologous DNA end-joining (NHEJ) and Rad51-related homologous recombinational repair (HRR); however, MGMT does not repair damages caused by tertiary bis(2-chloroethyl)amine drugs such as chlorambucil, melphalan, cyclophosphamide, ifosfamide, or DNA chelating agents such as platinum compounds [24]. Therefore, since tertiary bis(2-chloroethyl)amine drugs cross-link, rather than simply methylate DNA as TMZ does, they are more efficacious cytotoxic agents, and cells that are resistant to TMZ are frequently sensitive to tertiary bis(2-chloroethyl)amine drugs. Sensitivity to tertiary bis(2-chloroethyl)amine drugs is cell cycle-dependent; thus rapidly proliferating cells such as tumor cells are much more susceptible while quiescent cells such as the endothelial cells that form the BBB are not.

Most anticancer drugs that are used for systemic tumors are ineffective for treating CNS tumors, in large part due to their inability to cross the BBB and to enter the tumor and peri-tumoral areas at therapeutically meaningful concentrations. The BBB has low passive paracellular permeability and expresses high levels of active efflux drug transporters, which together limit brain exposure for many anticancer agents. Lack of BBB penetration has been an obstacle for many potential therapies for GBM. Direct intra-tumoral therapies via convection-enhanced delivery are currently being tested but have yet to show a survival benefit and have added risk of invasive surgery [25].

LAT1 is expressed at both the BBB and on tumor tissues; therefore, by combining LAT1 substrate activity with a potent tertiary bis(2-chloroethyl)amine cytotoxic moiety, the resulting chemotherapeutic molecule should be efficacious for treating TMZ-resistant gliomas. Melphalan is an amino acid analog of a tertiary bis(2-chloroethyl)amine similar to QBS10072S. However, it has failed in GBM clinical trials [26]. Due to its molecular architecture, specifically, substitution at the para-position of the tertiary N-(bis-(2-chloroethyl) amino group on the aromatic phenyl ring, melphalan is a poor substrate for LAT1 (Figure 1) [27]; therefore, only a fraction of the systemic melphalan is able to cross the BBB via LAT1 perhaps preventing enough chemotherapeutic agent to reach the peri-tumoral environment to be clinically effective. In addition, melphalan does not selectively interact with LAT1 and could enter normal brain cells via other transporters, such as LAT2, potentially leading to neurotoxicity and low tolerability. Therefore, a bifunctional tertiary bis(2-chloroethyl)amine that is a more selective, potent LAT1 substrate, such as QBS10072S, has the potential to be an effective treatment for GBM.

In this manuscript, we report the preclinical validation of QBS10072S as a novel chemotherapeutic agent with excellent BBB penetration and promising efficacy *in vitro* and *in vivo* against GBM, with the potential to be efficacious in patients regardless of MGMT status. The mechanism of action of QBS10072S is to cross-link DNA strands in highly proliferative cells, which inhibits the subsequent DNA replication necessary for cell growth and leads to apoptosis [22]. Since LAT1 is expressed on both sides of the BBB and the BBB cells are not highly proliferative, QBS10072S is transported across the BBB into the brain without damaging the BBB endothelial cells. Microscopic evaluation of brain tissue after dosing rodents with high levels of QBS10072S showed no alterations of the BBB suggesting that there was no damage to the microvessels, perhaps because these cells are not undergoing rapid proliferation and are not susceptible to DNA damaging agents. QBS10072S shows increased potency against high LAT1-expressing cells and causes dose-dependent DNA damage. QBS10072S is detectable at high levels in orthotopic GBM models with little accumulation in the surrounding normal brain and has limited toxicity in rodents. In two orthotopic mouse models of GBM, QBS10072S significantly inhibited tumor growth and prolonged survival. Importantly, QBS10072S combined with radiation to prolong median survival compared to the standard therapy of TMZ plus radiation.

Our *in vivo* preclinical validation studies were performed in orthotopic xenograft models that have the potential of a disrupted blood brain barrier by nature of the stereotactic injection used to create these xenografts. While we cannot eliminate the effect on the BBB in the models used for our studies, the results from a recent study with breast cancer metastatic tumors suggest that QBS10072S can cross the intact BBB to treat the tumor [28]. In this model, tumor cells are injected into the carotid artery, which then migrate to the leptomeninges to produce leptomeningeal metastatic disease. In this model, the BBB is never disrupted by the injection of tumor cells, and QBS10072S is still effective for inhibiting tumor growth.

Based on the unique expression of LAT1 on both the BBB and tumor cells and the potent anti-tumor activity of the tertiary bis(2-chloroethyl)amine class of chemotherapeutics, we have discovered a novel chemical entity (QBS10072S), which combines the molecular characteristics of a selective LAT1 substrate with the validated therapeutic properties of tertiary bis(2-chloroethyl)amine drugs. QBS10072S combines elements necessary for a successful brain tumor treatment: (1) selective transport by LAT1, conferring BBB penetration and uptake into cancer cells, (2) strong tumor cell cytotoxicity using a mechanism distinct from TMZ, and (3) low off-target cytotoxicity in normal brain, BBB, and other systemic organs at therapeutic doses. QBS10072S is a novel BBB permeable compound utilizing a clinically proven alkylating moiety that has the potential to transform the therapy for TMZ-resistant and relapsed/refractory GBM patients, for whom currently there are no other treatment options. QBS10072S is currently being studied in a phase one trial in refractory cancer patients, not limited to glioblastoma (ClinicalTrials.gov Identifier: NCT04430842).

## Conclusions

In this report, we identify a novel chemotherapeutic molecule with promising preclinical activity for the treatment of glioblastoma. By depending on a large amino acid transporter for cell penetration, this molecule has the potential to be delivered at high concentrations to brain tumors, with potentially decreased systemic toxicities compare to existing chemotherapeutic agents. A clinical trial in recurrent cancers is ongoing (ClinicalTrials.gov Identifier: NCT04430842).
